# Synthesis, Characterization, and Antiproliferative Properties of New Bio-Inspired Xanthylium Derivatives

**DOI:** 10.3390/molecules28031102

**Published:** 2023-01-22

**Authors:** Claudia Koch, Diana-Maria Dreavă, Anamaria Todea, Francisc Péter, Mihai Medeleanu, Iulia Păușescu, Corina Samoilă, Ioan Ovidiu Sîrbu

**Affiliations:** 1 Faculty of Industrial Chemistry and Environmental Engineering, Politehnica University Timişoara, Carol Telbisz 6, 300001 Timisoara, Romania; 2 Biochemistry Department, Faculty of Pharmacy, University of Medicine and Pharmacy “Victor Babeş”, Eftimie Murgu 2, 300041 Timisoara, Romania; 3 Center for Complex Networks Science, “Victor Babes” University of Medicine and Pharmacy, Eftimie Murgu 2, 300041 Timisoara, Romania

**Keywords:** xanthylium derivatives, halochromism, pH-jumps, monocarbonyl analogues of curcumin, antiproliferative activity, P19 cells

## Abstract

Xanthylium derivatives are curcumin analogs showing photochromic properties. Similarly, to anthocyanins, they follow the same multistate network of chemical species that are reversibly interconverted by external stimuli. In the present work, two new asymmetric monocarbonyl analogues of curcumin, 4-(4-hydroxy-3-metoxybenzylidene)-1,2,3,4-tetrahydroxanthylium chloride (compound **3**) and 4-(4-hydroxybenzylidene)-6-methoxy-1,2,3,4-tetrahydroxanthylium chloride (compound **4**) were synthesized, and their photochromic and biological properties were investigated. The UV-Vis spectroscopy and the direct and reverse pH-jumps studies confirmed the halochromic properties and the existence of different molecular species. A network of chemical reactions of these species was proposed. Furthermore, the antiproliferative properties of both compounds were evaluated using P19 murine embryocarcinoma cells and compared with each other. The results demonstrate that both new xanthylium derivatives modify the progression through the cell cycle of P19 cells, which translates into a significant antiproliferative effect. The effect of the methoxy group position is discussed and several checkpoint proteins are advanced as putative targets.

## 1. Introduction

Plants and plant extracts were from ancient times essential sources of bioactive compounds, particularly drugs, also becoming an important part of the medicine of our days [1,2]. The discovery of such bioactive molecules by screening and isolation from natural sources was accompanied by synthesis of derivatives or structural analogues, which also emerged as drug candidates, sometimes with even enhanced pharmacological effect especially for cancer and infectious diseases. These developments revitalized the interest for natural products as drug leads [3].

Among the bioactive components of plants, polyphenols are a diverse and multifunctional group with substantial health potential, including cancer prevention and treatment [4]. These health-promoting effects are linked to their antioxidant activity, leading to important antibiotic, anticancer, and anti-inflammatory properties. Polyphenols and their synthetic analogs interfere in carcinogenesis by modulating and regulating multiple signaling pathways and transcription factors, membrane-associated receptor tyrosine kinases, fatty acid metabolism and lipid rafts or methylation, together with other emerging targets [5]. Various synthetic analogues of natural polyphenols demonstrated strong anticancer activity, including (–)-epigallocatechin-3-gallate analogues [6], synthetic anthocyanidins [7], or cinnamic acid derivatives [8]. Some analogues with known anticarcinogenic effect proved even higher stability to metabolic conversion and displayed comparable or higher antitumor activity than the parent compound [9].

Curcumin is a polyphenolic curcuminoid extracted from the roots of turmeric (*Curcuma longa* L.), cultivated in tropical and subtropical regions for food coloring or spice use. Due to its many therapeutic properties (e.g., antiseptic, antiproliferative, antioxidant and anti-inflammatory), curcumin is also used as a herbal remedy for the treatment of a wide range of diseases such as infections, injuries, arthritis, pancreatitis [10,11,12], diabetes or diabetic cardiomyopathy [13]. Curcuminoids content of turmeric is 2–9%, and include a group of compounds such as curcumin, *bis*-demethoxycurcumin, demethoxycurcumin, and cyclic curcumin [14]. Numerous studies have highlighted the anticarcinogenic effect of curcumin and the mechanism by which it inhibits cell growth and proliferation and stimulates the apoptosis of cancer cell lines in vitro [15,16,17]. Recent studies have proved that curcumin can obstruct the differentiation of cancer stem cells and their self-renewal and can also inhibit tumor cells to blood vessels [17].

To reduce the major disadvantages of curcumin (e.g., low bioavailability and rapid metabolism) various strategies have been proposed, such as the development of curcumin adjuvants, curcumin nanoparticles, or synthetic analogues of curcumin [14]. During the last few years, synthetic modifications of curcumin have focused on replacing the β-diketone fragment with a monocarbonyl spacer, which aimed to reduce the instability of curcumin and improve its bioactivity [12,18].

2,6-*bis*-(Arylidene)-cyclohexanones are such a class of curcumin analogs that gained increasing scientific interest. A hydroxyl group inserted in the ortho position of at least one of the arylidene fragments led to a new class of multistate molecules. This recently emerged family of compounds exhibited photochromic properties following a similar multistate network of chemical reactions as anthocyanins. They can be obtained in the open (curcumin type) or the closed (anthocyanin/xanthylium derivatives type) forms and can be reversibly switched from one species to another when are subjected to different stimuli like pH changes, light, or temperature [19,20,21].

This work addresses the synthesis and characterization of two new synthetic bio-inspired xanthylium salts, holding methoxy substituents in the aromatic rings. The synthesized compounds, structure, and purity were assessed based on FT-IR and NMR spectroscopy. The halochromic properties and time stability of the synthesized salts were investigated by UV-Vis spectroscopy and pH-jumps study, highlighting the reversibility of the complex multistate network of species. Furthermore, the anticancer properties of the synthesized compounds were evaluated on P19 carcinoembryonic cells, demonstrating their antiproliferative effect. P19 is a pluripotent murine embryonic teratocarcinoma cell line, which possess the ability to differentiate into cells from all three germ layers [22]. The treatment of P19 cells to retinoic acid leads to the formation of neurons, while exposure to dimethyl sulfoxide (DMSO), leads to cardiac or skeletal muscle cells [23]. Since P19 cells are easier to maintain in culture than other embryonic stem cells, they are a valuable model for in vitro cell differentiation studies. Techniques for manipulating this cell line allow a detailed investigation of signaling pathways, cell cycle differentiation and regulation mechanisms and gene expression regulation [24]. These promising results allow further investigations in this class of curcumin analogues with anticancer effect, aiming their microencapsulation and targeted delivery.

## 2. Materials and Methods

### 2.1. Materials

4-Hydroxy-3-methoxybenzaldehyde, 2-hydroxy-4-methoxybenzaldehyde (98%), salicylaldehyde (98%), 4-hydroxy-benzaldehyde (98%), cyclohexanone (99%), boric acid (H_3_BO_3_, >99.5%), citric acid (>99.5%) trisodium phosphate (Na_3_PO_4_, 96%) and sodium hydroxide (NaOH, 99%) were acquired from Sigma Aldrich (Steinheim am Albuch, Germany). Methanol (MeOH, >99.9%) and sulphuric acid (H_2_SO_4_, 95–97%) were purchased from CHIMREACTIV SRL (Bucuresti, Romania) and Merck KGaA (Darmstadt, Germany). All reagents and solvents used in the experiments were used without further purification.

Fetal bovine serum (FBS), Dulbecco’s phosphate-buffered saline (DPBS without Ca and Mg), Trypsin, Minimum Essential Medium α (α-Mem), Roswell Park Memorial Institute (RPMI) 1640 Medium with Glutamax, and Penicillin-Streptomycin (Penstrep) were acquired from Gibco ThermoScientific LSG (Auckland, New Zealand). Dimethylsulfoxide was purchased from Fluka Analytical (Seelze, Germany). Muse System Check Kit, Muse Count & Viability Kit and Muse Cell Cycle Kit were acquired from Merck KGaA (Darmstadt, Germany).

### 2.2. Methods

#### 2.2.1. Synthesis and Characterization of the New Xanthylium Derivatives

The intermediate compounds, 2-(2-hydroxy-benzylidene)-cyclohexane-1-one (compound **1**) and 2-(4-hydroxy-benzylidene)-cyclohexane-1-one (compound **2**) were obtained by condensation reaction of 0.05 moles of a specific benzaldehyde (salicylaldehyde and 4-hydroxy-benzaldehyde, respectively) with cyclohexanone (25.9 mL, 0.25 moles), as previously described [25].

The synthesis of compounds **3** and **4** was accomplished by a condensation reaction under acidic conditions, following the procedure of Pana et al. [26].

0.002 moles of the appropriate ketone (compound **1** or **2,** respectively) and 0.002 moles of suitable benzaldehyde (vanillin for compound **3** and 2-hydroxy-4-methoxy-benzaldehyde for compound **4**) were dissolved in 40 mL methanol at room temperature, under magnetic stirring. Hydrochloric acid gas generated in situ in an attached installation was bubbled in the solution under continuous stirring. After about 10 min from the start of the bubbling, the color of the solution turned from yellow-orange to red-cherry. After 1 h, the color of the solution became intense cherry, and a precipitate appeared. After 1.5 h, the bubbling with hydrochloric acid was stopped and the reaction mixture was left to stir at room temperature for another 1 h. The precipitate was separated by filtration, dried at room temperature for five days, resulting in 458 mg of dark green precipitate (compound **3**) and 310 mg of dark brown precipitate (compound **4**).

**2-(2-Hydroxy-benzylidene)-cyclohexan-1-one (1)**

1136 mg of yellow precipitate, yield 11.3%, m.p. = 126–128 °C

FTIR (ATR) cm^−1^: 3363 ($\nu_{OH}$), 2939 ($\nu_{CH2}^{as}$), 1655 ($\nu_{C=O}$), 1601 (ν_ar. sk „1600”_), 1560 (ν_ar. sk „1600”_), 1450 (ν_ar. sk „1500”_), 1256 ($\nu_{C-O}$), 750 (1,2-disubstituted benzene ring) (Supplementary Materials, Figure S1).

^1^H NMR (300 MHz, CD_3_OD, δ ppm): 7.64 (s, 1H, H7), 7.24 (d, *J* = 7.7 Hz, 1H, H13), 7.16 (dd, *J* = 11.6, 3.9 Hz, 1H, H11), 6.83 (dd, *J* = 5.5, 3.8 Hz, 2H, H10, H12), 2.76 (td, *J* = 6.5, 2.1 Hz, 2H, H6), 2.50 (t, *J* = 6.7 Hz, 2H, H3), 1.92 (dt, *J* = 12.9, 6.5 Hz, 2H, H5), 1.75 (dd, *J* = 12.1, 5.9 Hz, 2H, H4) (Supplementary Materials, Figure S5).

^13^C-NMR (75 MHz, CD_3_OD, δ ppm): 204.6 (C1), 158.1 (C9), 137.4 (C2), 133.2 (C7), 131.5 (C11, C13), 123.9 (C8), 120.1 (C12), 116.5 (C10), 41.4 (C6), 30.2 (C3), 25.1 (C4), 24.7 (C5) (Supplementary Materials, Figure S6).

**2-(4-Hydroxy-benzylidene)-cyclohexan-1-one (2)**

1180 mg of yellow-green precipitate, yield 11.7%, m.p. = 171–173 °C

FTIR (ATR) cm^−1^: 3207 ($\nu_{OH}$), 2933 ($\nu_{CH2}^{as}$), 1653 ($\nu_{C=O}$), 1549 (ν_ar. sk „1600”_), 1429 (ν_ar. sk „1500”_), 1271 ($\nu_{C-O}$), 835 (1,4-disubstituted benzene ring) (Supplementary Materials, Figure S2).

^1^H NMR (300 MHz, CD_3_OD, δ ppm): 7.44 (s, 1H, H7), 7.34 (d, *J* = 8.7 Hz, 2H, H3, H5), 6.82 (d, *J* = 8.7 Hz, 2H, H2, H6), 2.83 (td, *J* = 6.6, 2.1 Hz, 2H, H10), 2.47 (t, *J* = 6.7 Hz, 2H, H13), 1.89 (dd, *J* = 12.2, 6.0 Hz, 2H, H11), 1.76 (dd, *J* = 11.5, 6.2 Hz, 2H, H12) (Supplementary Materials, Figure S7).

^13^C-NMR (75 MHz, CD_3_OD, δ ppm): 204.0 (C9), 160.0 (C1), 138.1 (C7), 134.7 (C8), 133.9 (C3, C5), 128.2 (C4), 116.5 (C2, C6), 41.0 (C13), 30.1 (C10), 24.9 (C12), 24.3 (C11) (Supplementary Materials, Figure S8).

**4-(4-Hydroxy-3-metoxybenzylidene)- 1,2,3,4-tetrahydroxanthylium chloride (3)**

458 mg of dark green precipitate, yield 72.5%, m.p. = 155–157 °C

FT-IR (ATR) cm^−1^: 3367 ($\nu_{OH}$), 2987 ($\nu_{CH3}^{as}$), 1574 (ν_ar. sk „1600”_), 1491 (ν_ar. sk „1500”_), 1410 (ν_ar. sk „1500”_), 1242 (ν_Car-O_), 1130 ($\nu_{CH3}^{s}$_-O_), 825 (1,2,4- trisubstituted benzene ring) (Supplementary Materials, Figure S3).

^1^H NMR (500 MHz, CD_3_OD, δ ppm): 8.78 (s, 1H, H14), 8.63 (s, 1H, H7), 8.19 (d, *J* = 8.6 Hz, 1H, H19), 8.09 (dd, *J* = 13.9, 6.9 Hz, 2H, H16, H18), 7.79 (t, *J* = 7.6 Hz, 1H, H17), 7.51 (dd, *J* = 8.4, 1.7 Hz, 1H, H13), 7.44 (d, *J* = 1.7 Hz, 1H, H9), 6.95 (d, *J* = 8.4 Hz, 1H,12), 3.92 (s, 3H, H21), 3.14 (t, *J* = 5.6 Hz, 2H, H3), 3.12–3.09 (m, 2H, H5), 2.07–2.02 (m, 2H; H4) (Supplementary Materials, Figure S9).

^13^C NMR (125 MHz, CD_3_OD, δ ppm): 174.6 (C1), 156.6 (C20), 154.8 (C11), 151.4 (C14), 151.2 (C7), 149.7 (C10), 138.4 (C18), 133.6 (C6), 131.3 (C13), 130.9 (C16), 130.3 (C17), 129.1 (C8), 126.6 (C2), 125.3 (C15), 119.6 (C19), 117.8 (C9), 117.6 (C12), 56.8 (C21), 29.4 (C3), 28.8 (C5), 22.07 (C4) (Supplementary Materials, Figure S10).

**4-(4-Hydroxybenzylidene)-6-methoxy-1,2,3,4-tetrahydroxanthylium chloride (4)**

310 mg of dark brown precipitate, yield 48.7%, m.p. = 149–151 °C

FT-IR (ATR) cm^−1^: 1622 (ν_ar. sk „1600”_), 1572 (ν_ar. sk „1600”_), 1491 (ν_ar. sk „1500”_), 1439 (ν_ar. sk „1500”_), 1238 (ν_Car-O_), 1146 ($\nu_{CH3}^{s}$_-O_), 839 (1,4- disubstituted benzene ring) (Supplementary Materials, Figure S4).

^1^H NMR (500 MHz, DMSO-*d_6_*, δ ppm): 9.01 (s, 1H, H7), 8.49 (s, 1H, H15), 8.15 (d, *J* = 9.0 Hz, 1H, H3), 7.98 (s, 1H, H6), 7.77 (d, *J* = 8.8 Hz, 2H, H17, H21), 7.51 (d, *J* = 9.0 Hz, 1H, H2), 7.04 (d, *J* = 8.7 Hz, 2H, H18, H20), 4.09 (s, 3H, H23), 3.03 (dd, *J* = 12.5, 6.5 Hz, 4H, H11, H13), 1.97–1.91 (m, 2H, H12) (Supplementary Materials, Figure S11).

^13^C NMR (125 MHz, DMSO-*d_6_*, δ ppm): 169.3 (C9), 166.8 (C1), 161.2 (C19), 157.1 (C5), 150.6 (C7), 143.6 (C15), 134.6 (C17, C21), 130.6 (C3), 127.8 (C8), 125.6 (C16), 124.2 (C14), 120.0 (C2), 118.5 (C40, 115.9 (C18, C20), 99.7 (C6), 56.7 (C23), 26.6 (C13), 26.3 (C11), 19.9 (C12) (Supplementary Materials, Figure S12).

#### 2.2.2. UV-Vis and FT-IR Spectroscopy

UV-Vis absorption spectra were registered at 20 °C using an Agilent Cary 60 spectrophotometer (Agilent Technologies, Waldbronn, Germany).

FT-IR spectra were collected on a Bruker Vertex 70 (Bruker Daltonik GmbH, Bremen, Germany) spectrometer connected with a Platinium ATR, Bruker Diamond Type A225/Q. The samples spectra were collected after 64 co-added scans, on a spectral domain of 4000–400 cm^−1^, with a resolution of 4 cm^−1^.

#### 2.2.3. NMR Analysis

NMR spectra were collected on a Bruker AVANCE III spectrometer (Bruker Daltonik GmbH, Bremen, Germany) working at 500.0 MHz (^1^H) and 125.0 MHz (^13^C) at 298 K. Chemical shifts δ are reported in ppm versus tetramethylsilane, TMS, coupling constants are reported in Hz and for splitting pattern the following abbreviations are used: s (singlet), d (doublet), t (triplet), dd (doublet of doublets), td (triplet of doublets), dt (doublet of triplets) and m (multiplet). The samples were dissolved in DMSO-*d_6_* or CD_3_OD. NMR assignments have been performed based on 1D NMR spectra (^1^H, ^13^C, DEPT 135) and 2D NMR spectra (COSY, HQSC, HMBC) analysis.

#### 2.2.4. Study of pH Dependent Photochromic Properties

The halochromic properties study of the synthesized xanthylium dyes involved spectrophotometric monitoring of color variation of dye solutions at different pH values over time. The buffer solutions in the pH range from 2 to 12 were prepared based on boric acid, citric acid and trisodium phosphate solutions following a previously described procedure [27].

#### 2.2.5. Biological Activity Study

The P19 cells were cultured at 37 °C and 5% CO_2_ atmosphere in a SANYO MCO-5AC incubator. Cell quantification and cell cycle was studied with the Muse Cell Analyzer microflow-cytometer using dedicated reagent kits: Muse System Check Kit (MCH100101) for device calibration, Muse Count and Viability Kit (MCH100102) for live cell counting and Muse Cell Cycle Kit (MCH100106) for aggregate cell cycle analysis.

## 3. Results and Discussions

### 3.1. Synthesis and Characterization of the Xanthylium Dyes

**Figure 1:**
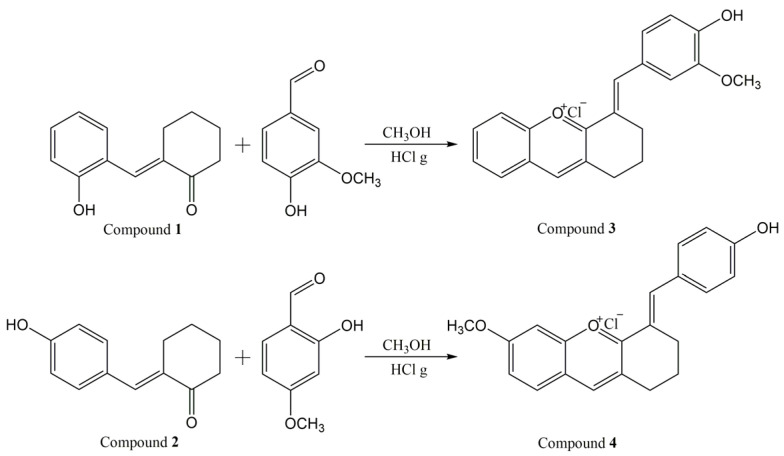
Reaction scheme for the synthesis of the xanthylium salts **3** and **4**.

Two new synthetic bio-inspired xanthylium salts, obtained in a closed form, were synthesized, and characterized. Figure 1 depicts the reaction scheme for the synthesis of compounds **3** and **4**.

The structure and the purity of the synthesized salts were assessed and demonstrated based on FT-IR, 1D and 2D NMR spectra.

The FT-IR analysis confirmed the presence of the main functional and structural groups in all the synthesized compounds as presented in Section 2.2.1.

**Figure 2:**
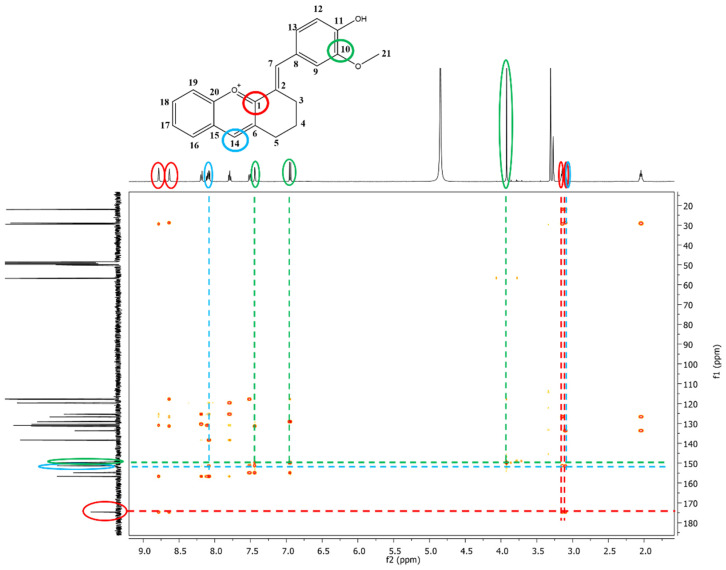
The ^1^H-^13^C-HMBC spectrum of compound **3**—the remote couplings between carbon atoms and protons are depicted by different colored circles, green—C10 with H9, H12, and H13, red—C1 with H3, H5, H7, and H14, blue—C14 with H5, and H16.

The exact structures of the synthesized dyes were demonstrated by NMR analysis. In the ^1^H-NMR spectrum of compound **3**, the multiplets at 2.07–2.02 ppm, 3.12–3.09 ppm and the signals at 3.14 and 3.92 ppm, which correspond to aliphatic protons were attributed to the methylene protons from the cyclohexanone moiety (-CH_2_) and to the methoxy group (-OCH_3_) from vanillin. The aromatic protons showed signals between 6.95 and 8.19 ppm. The most deshielded signals were found for protons in CH groups due to the extended π conjugation of the molecule (H7 and H14, Supplementary Materials, Figure S5). The signals at 22.0, 28.8, and 29.4 ppm in the ^13^C-NMR spectrum of compound **3** correspond to the carbon atoms of the three methylene groups (-CH_2_) present in the structure. Another aliphatic signal was identified at 56.8 ppm and attributed to the carbon atom from the methoxy group (-OCH_3_). The aromatic carbon atoms were assigned to signals between 117.6 and 156.6 ppm. The carbon atoms linked to oxygen atoms were found to be the most deshielded ones with signals at 174.1 ppm (C=O^+^), 156.6 ppm (C-O^+^), 154.8 (C-OH), 149.7 ppm (C-OCH_3_). Additional proof of the formation of compound **3** was given by the remote couplings between carbon atoms and protons, shown in the two-dimensional HMBC spectrum (Figure 2). The signal corresponding to C1 carbon atom (174.5 ppm) coupled over two bonds with protons H3 (3.14 ppm), H5 (3.12–3.09 ppm), H7 (8.63 ppm) and H14 (8.78 ppm). It can also be observed the remote coupling of C10 (149.7 ppm) with three protons: H23 (3.92 ppm), H9 (7.44 ppm) and H12 (6.95 ppm). The remote coupling over two bonds of C14 carbon atom (151.4 ppm) with protons H5 (3.12–3.09 ppm) and H16 (8.09 ppm) was a further confirmation of the formation of compound **3.**

Since the only structural difference between compounds **3** and **4** is the position of the methoxy group for compound **4** the characterization details are presented in the Supplementary Materials.

### 3.2. pH-Dependent Photochromic Properties

The halochromic properties of the synthesized salts were investigated by UV-Vis spectroscopy.

**Figure 3:**
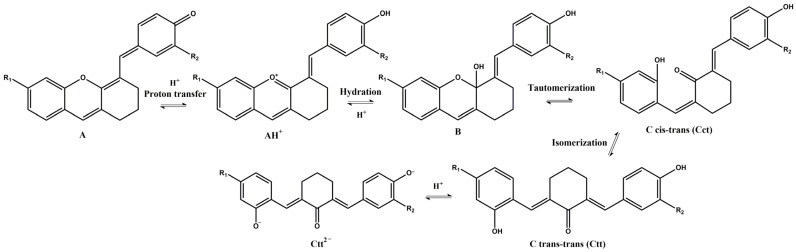
Potential network of chemical reactions involved in the interconversion of the species for compound **3** (R_1_ = H; R_2_ = OCH_3_) and compound **4** (R_1_ = OCH_3_; R_2_ = H).

Similar to the anthocyanins, these xanthylium derivatives show photochromic properties and follow the same multistate network of chemical species, which can reversibly pass from one species to another when are subjected to the action of different stimuli like pH changes, temperature, or light [20]. Figure 3 depicts a potential network of chemical reactions involved in the interconversion of the species under acidic and basic conditions.

**Figure 4:**
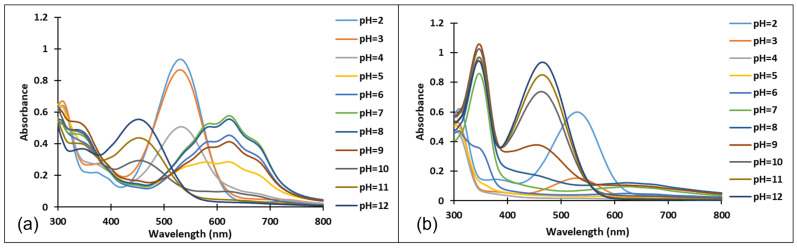
Absorption spectra of the species derived from compound **3,** after (**a**) 40 min and (**b**) 72 h, at different pH values.

**Figure 5:**
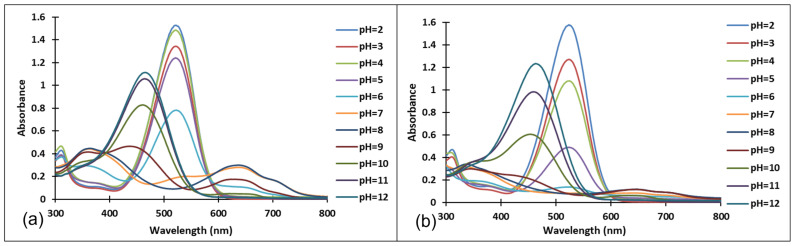
Absorption spectra of the species derived from compound **4** after (**a**) 40 min and (**b**) 72 h at different pH values.

The existence of these species was confirmed by the UV-Vis spectra (Figure 4 and Figure 5). It should be noted that the structural differences between compounds **3** and **4**, namely the different positioning of the methoxy group on the aromatic ring, did not significantly influence the halochromic properties and the corresponding species exhibit almost similar absorption bands.

Since the synthesis of compounds **3** and **4** was accomplished in acidic medium, the purple-colored xanthylium cations AH^+^ (compound **3**: λ = 539 nm; compound **4**: λ = 521 nm) were obtained. The AH^+^ species were transformed into blue-colored quinoidal bases (A) (compound **3**: λ = 624 nm with two shoulders around 586 nm and 670 nm; compound **4**: λ = 625 nm), by a proton transfer process. When hydrating the AH^+^ cations, colorless hemiketals (B) are obtained, which upon increasing the pH, by opening the cycle, convert to the *cis* and *trans* chalcones (Cct, Ctt), yellow-colored neutral species (compound **3**: λ = 345 nm; compound **4**: λ = 363 nm). The orange-colored ionized species (Ctt^2−^) (compound **3**: λ = 459 nm; compound **4**: λ = 464 nm) are obtained by deprotonation of the hydroxyl groups in basic medium.

Figure 4 and Figure 5 show the absorption spectra of compounds **3** and **4**, collected after 40 min and 72 h at different pH values. It must be specified that the AH^+^ species corresponding to the strong acid medium (pH = 2) and those corresponding to strong basic medium Ctt^2−^ (pH = 12) are stable in time at room temperature, so that they can be stored for long periods of time.

**Figure 6:**
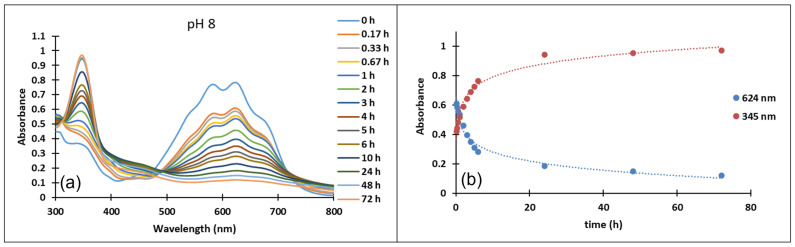
Absorption spectra of the species derived from compound **3** (**a**) at pH 8 in time (**b**) variation in time of the absorption maxima of the quinoidal base A λ = 624 nm and the *cis* or *trans* chalcones λ = 345 nm at pH 8.

In the case of compound **3**, in the acidic pH domain (2–4) increasing the pH leads to the bleaching of the solutions in time, corresponding to the formation of a hemiketal (B). In the pH range 5 to 6, the predominant species is the quinoidal base (A). At pH values 7 and 8, the formation of the neutral species Cct, Ctt can be observed (Figure 6). The deprotonated forms of the neutral species appear in the pH range 9 to 12. The quinoidal base (A) was observed only until the pH reached the value 10, above which the solutions become orange and exhibit two absorption bands at 345 nm and 459 nm, corresponding to the neutral and deprotonated species.

In the pH range 3 to 6, compound **4** is not stable in time and bleaching of the solutions was observed, corresponding to the formation of the hemiketal (B). In the pH range 7 to 9 the predominant species are the quinoidal base (A) and *cis* and *trans* chalcones (Cct, Ctt), which are not stable over time.

**Figure 7:**
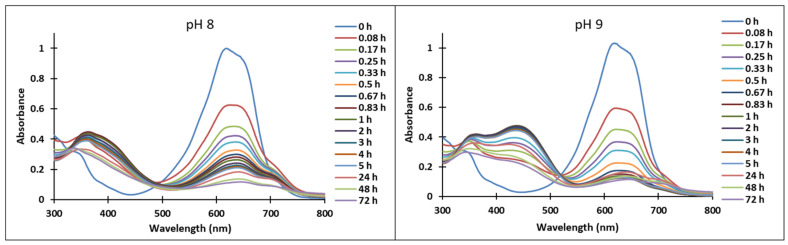
Absorption spectra of the species derived from compound **4** in time at pH 8, respectively pH 9.

The transformations of species for compound **4** over time are shown in Figure 7. At pH 8, the formation of neutral species Cct, Ctt from the quinoid base (A) was noticed, while at pH 9 the formation of the species (Ctt^2−^) was observed.

### 3.3. pH-Jumps Study

To investigate the reversibility of the multistate network of species, direct and reverse pH-jumps were performed. A direct pH-jump involves the addition of base to solutions equilibrated in a strongly acidic medium, while a reverse pH-jump corresponds to the addition of acid to solutions stable at higher pH values.

**Figure 8:**
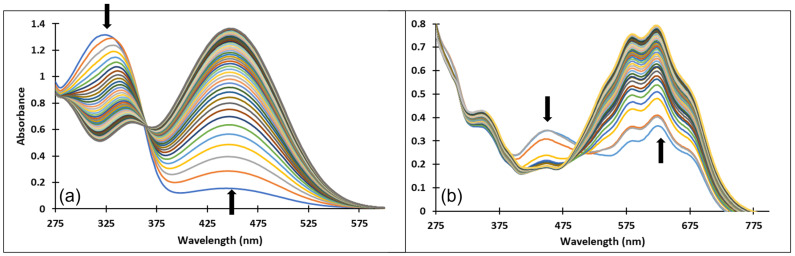
Spectral variations of a solution of compound **3** after: (**a**) direct pH-jump from pH 2.07 to pH 12.13 and (**b**) reverse pH-jump from pH 12.06 to pH 7.23.

Upon a direct pH-jump from an equilibrated solution of compound **3**, at pH = 2.07 to pH = 12.13, only the formation of the deprotonated species was observed (Figure 8a). The transition from the AH^+^ species to the quinoidal base (A) is achieved through a proton transfer, which is the fastest kinetic process that occurs and can be captured spectrophotometrically only with a stopped-flow system.

The spectral variations accompanying a reverse pH-jump of an equilibrated solution of compound **3** from pH = 12.06 to pH = 7.23 leading to the formation of the neutral species *cis* and *trans* chalcones (Cct, Ctt) and the quinoidal base (A) are shown in Figure 8b).

**Figure 9:**
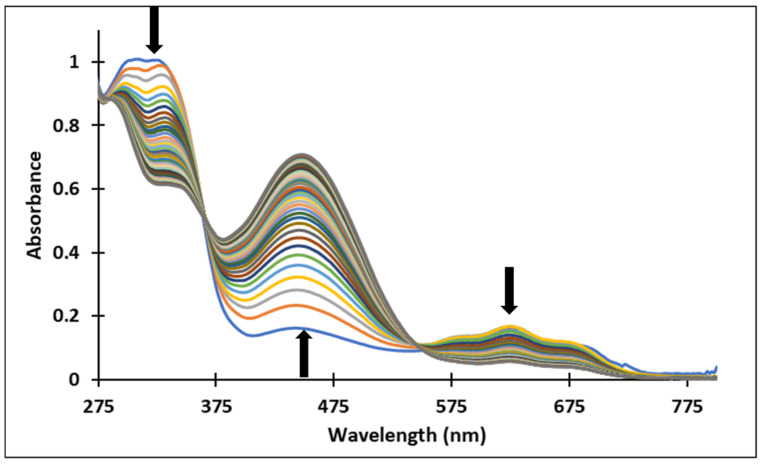
Spectral variations after pH-jump of a solution of compound **3**, from pH 6.09 to pH 12.16.

The formation of the deprotonated species (Ctt^2−^, λ = 459 nm) from the quinoidal base (A, λ = 624 nm with two shoulders around 586 nm and 670 nm) was evidenced also by the direct pH-jump of a solution of compound **3** equilibrated from pH 6.09 to 12.16 (Figure 9).

**Figure 10:**
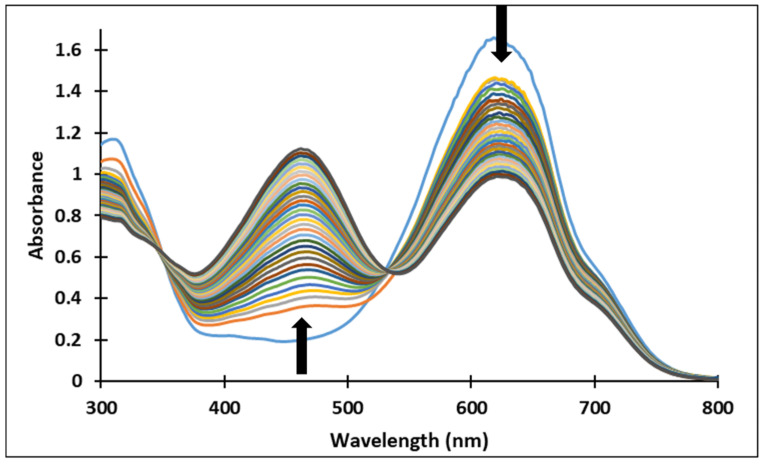
Spectral variations after direct pH-jump of solution of compound **4**, from pH 2.09 to pH 9.72.

Figure 10 presents the spectral variations that occur upon a direct pH-jump of an equilibrated solution of compound **4** from pH = 2.09 to pH = 9.72 showing the formation of the deprotonated species (Ctt^2−^, λ = 459 nm) from the quinoidal base (A, λ = 625 nm). The transformation from the AH^+^ specie to the quinoidal base (A) was not captured because the proton transfer is an extremely fast kinetic process.

**Figure 11:**
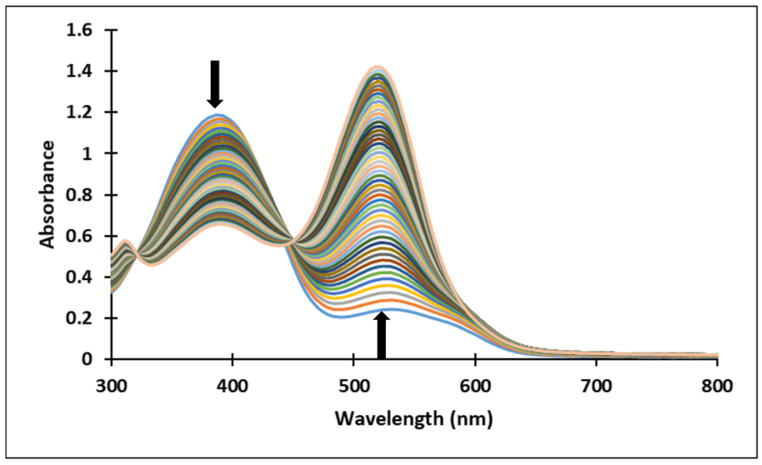
Spectral variations after direct pH-jump of solution of compound **4**, from pH 12.17 to pH 0.12.

Figure 11 shows the spectral variations accompanying a reverse pH-jump of an equilibrated solution of compound **4** with pH = 12.17 to pH = 0.12 as a result of the formation of the xanthylium cation AH^+^ with an absorption band at 521 nm from the ionized specie (Ctt^2−^) (λ = 464 nm).

**Figure 12:**
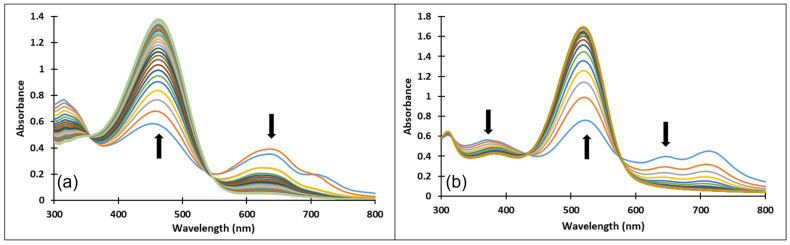
Spectral variations after direct pH-jumps of compound **4** solution, from (**a**) neutral to basic or (**b**) neutral to acidic pH values.

Upon a direct pH-jump from an equilibrated neutral solution of compound **4** at pH = 6.58 to pH = 9.94 (Figure 12a) the decrease of the absorption band corresponding to the quinoidal base (A) at 625 nm and the formation of the deprotonated species (Ctt^2−^) at 464 nm were observed. The reverse pH-jump of an equilibrated neutral solution at pH = 7.01 to pH = 2.39 (Figure 12b), resulted in the decrease of the specific absorption bands for neutral species (A) at 625 nm, and *cis* and *trans* chalcones (Cct, Ctt) at 363 nm along with the increase of the absorption band corresponding to the xanthylium cation AH^+^ at 521 nm.

### 3.4. Inhibition of Cell Proliferation

The characterization of the biological activity of the compounds **3** and **4** was performed on P19 embryonic teratocarcinoma cells and involved the determination of the antiproliferative concentrations and the effect on cell cycle progression. P19 cells are frequently used for the evaluation of cytotoxicity and the impact on the cell cycle of synthetic or natural compounds [28,29], and were adopted it in our study as an ex vivo model for screening for antiproliferative effects, due to their ease of maintenance in culture and high proliferation rate.

Table 1 shows the arithmetic mean, the final number of cells, the statistical significance of the obtained results, and the percentage of proliferation inhibition for the two compounds.

The IC_50_ values of compounds **3** and **4** are 233.0544 µM and 98.3751 µM, respectively (calculated using an online resource [30]).

**Figure 13:**
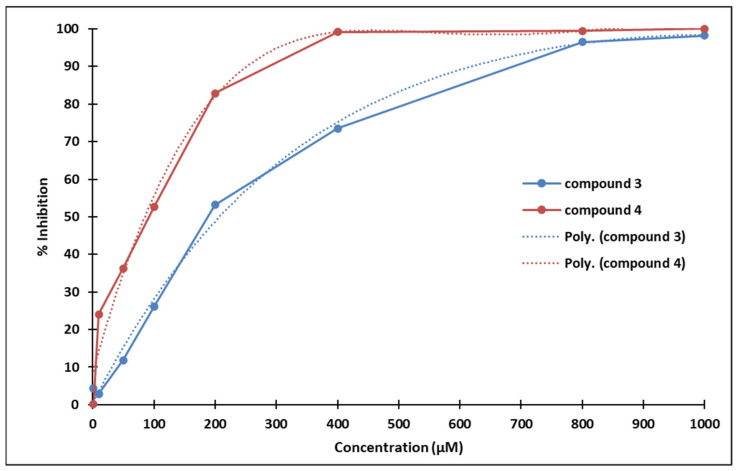
Dose-dependent inhibition of P19 cells exposed to compounds **3** (blue) and **4** (red).

Compound **3** strongly inhibits P19 cells proliferation starting with concentrations above 100 µM (statistical significance threshold *p* = 0.05). Similarly, compound **4** also inhibits P19 cells proliferation starting with concentrations above 50 µM (statistical significance threshold *p* = 0.05). The ostensibly more pronounced antiproliferative effect of compound **4** compared to **3** might be due to the different positioning of the methoxy group (Figure 13).

Poly (**3**): y = −9 × 10^−11^x^4^ + 3 × 10^−7^x^3^ – 0.0004x^2^ + 0.3155x + 0.5655; R² = 0.9955

Poly (**4**): y = −5 × 10^−10^x^4^ + 1 × 10^−6^x^3^ – 0.0014x^2^ + 0.6012x + 8.4036; R² = 0.9826

### 3.5. The Effect on Cell Cycle Progression

To further dissect the biological activity of the new xanthylium derivatives, their influence on cell cycle progression were analyzed, selecting two concentrations close to IC_50_ values and at which the proliferation inhibition effect is comparable (around 50%): 200 µM for compound **3**, and 100 µM for compound **4**.

Table 2 shows the percentage of P19 cells in each phase of the cell cycle and the analysis of statistical significance. Compound **3** produces a significant accumulation of the cell population in the G2/M phase (with a corresponding decrease of distribution in G0/G1 and S phases), indicating a possible blockage of progression to or through mitosis by action at the level of G2 and/or M checkpoints.

As expected, the P19 cells exposed to compound **4** show a similar cell cycle distribution profile with a significant accumulation in G2/M phase and a decrease of the number cells in G0/G1 and S phases. This suggests that compound **4** also impacts the activity of the proteins involved in G2 and M checkpoints (Cyclin A, Cyclin B, CDK1, etc.). Although the cell cycle redistribution in G2/M phase is similar for the two compounds, there are significant changes in G0/G1 and S phase cell numbers. Nevertheless, the overall distribution is similar, with preponderance of cells in the G2/M phase, indicating similar mechanisms of action.

The mechanisms underlying these effects involve most probably impact on the control of the G2/M checkpoint and on the pro-apoptotic mechanisms. From this perspective, these results are well in line with published data exploring antiproliferative and pro-apoptotic effects of curcumin derivatives on cancer cells [14]. Vero et al. [31] described a strong cytotoxic effect of similar asymmetrical mono-carbonyl curcumin analogues on Vero, HeLa, and MCF7 cells, with LC_50_ and IC_50_ values in the micromolar range similar to the ones determined in our experiments. Qiu et al. [12] reported a significant, dose-dependent G2/M arrest and apoptosis of cholangiocarcinoma cell lines exposed to allylated mono-carbonyl curcumin analogues presumably through p53/Cdc2 and Bax/Bcl-2 signaling, respectively. Similar effects were described by Xia et al. [32] on human gastric cancer cell lines exposed to asymmetric mono-carbonyl analog of curcumin. Overall, it appears that the G2/M blockage and induction of apoptosis are general features of curcumin derivatives, the variation in biological activities being given by the nature and position of the different side groups.

## 4. Conclusions

Two new synthetic bio-inspired xanthylium salts were synthesized and fully characterized. By performing a UV-Vis spectroscopy study, and direct and reverse pH-jumps, the halochromic properties and the reversibility of the multistate network of species were explored. A network of chemical reactions of the species was also proposed.

Both compounds **3** and **4** have a significant biological antiproliferative activity on P19 embryocarcinoma cells, with IC_50_ values around 100 µM for compound **4** and 200 µM for compound **3**. For the time being, we do not know whether this effect is due only to the inhibition of cell cycle progression or it also associates a pro-apoptotic effect.

The position of the methoxy group does not seem to alter the overall biological effect of the two xanthylium derivatives: the inhibition of cell proliferation through blockage of G2/M progression. Nevertheless, the different positions of the methoxy group might be responsible for the changes observed in G0/G1 and S phase distribution. Furthermore, the position of the methoxy group might also significantly influence the compound concentration at which the inhibitory effect becomes significant: 50 µM for compound **4** and 100 µM for compound **3**.

Further experiments will be performed to elucidate the interactions of these molecules, the role of positioning the methoxy group in benzene cycles in modulating these interactions and the spectrum of intracellular signaling changes caused by exposure to these molecules.

## Figures and Tables

**Table 1:** Dose-dependent inhibition of P19 cells exposed to compounds **3** and **4** (Student *t*-test, 2 tails, heteroscedastic).

	Compound 3		Compound 4	
Concentration (µM)	% Inhibition	*t*-Test	% Inhibition	*t*-Test
1000	98.19	6.49 × 10^−2^	100	8.97 × 10^−3^
800	96.51	1.72 × 10^−4^	99.45	1.82 × 10^−2^
400	73.51	2.38 × 10^−2^	99.22	7.68 × 10^−2^
200	53.14	3.23 × 10^−2^	82.85	1.83 × 10^−3^
100	26.03	4.25 × 10^−1^	52.59	3.48 × 10^−2^
50	11.84	6.53 × 10^−1^	36.33	9.40 × 10^−5^
10	2.86	8.47 × 10^−1^	24.11	1.08 × 10^−1^
1	4.29	8.29 × 10^−1^	0.16	9.92 × 10^−1^

**Table 2:** Cell phase distribution (%) of P19 cells upon exposure to compounds **3** and **4** (Student *t*-test. 2 tails. heteroscedastic).

	Control	Compound 3		Compound 4		3 vs. 4
		%	*t*-Test	%	*t*-Test	*t*-Test
G0/G1	48.38	40.73	6.45 × 10^−3^	35.70	7.87 × 10^−4^	8.94 × 10^−4^
S	19.12	17.72	7.83 × 10^−2^	22.55	1.55 × 10^−2^	1.74 × 10^−4^
G2/M	32.42	41.43	1.98 × 10^−5^	41.63	2.28 × 10^−5^	5.91 × 10^−1^

## Data Availability

Not applicable.

## Supplementary Materials

The following supporting information can be downloaded at: https://www.mdpi.com/article/10.3390/molecules28031102/s1, Figure S1: FT-IR spectra of compound **1**, Figure S2: FT-IR spectra of compound **2**, Figure S3. FT-IR spectra of compound **3**, Figure S4: FT-IR spectra of compound **4**, Figure S5: 1H NMR spectra of compound **1**, Figure S6: 13C NMR spectra of compound **1**, Figure S7: 1H NMR spectra of compound **2**, Figure S8: 13C NMR spectra of compound **2**, Figure S9: 1H NMR spectra of compound **3**, Figure S10: 13C NMR spectra of compound **3**, Figure S11: 1H NMR spectra of compound **4**, Figure S12: 13C NMR spectra of compound **4**, Figure S13: The 1H-^13^C-HMBC spectrum of compound **4**—the remote couplings between carbon atoms and protons are depicted by different colored circles, green—C5 with H3 and H7, red—C9 with H7, H11, H13, and H15, blue—C15 with H13, H17, and H21.
