# Supplementary material for: Synthesis, Characterization, and Antiproliferative Properties of New Bio-Inspired Xanthylium Derivatives
Source: Molecules. 2023 Jan 22;28(3):1102. doi: 10.3390/molecules28031102 (PMC9921969; doi:10.3390/molecules28031102)
Supplement: Supplementary file 1 [file molecules-28-01102-s001.zip › molecules-2144471-supplementary.pdf]

Synthesis, characterization, and anticancer properties of new bio-inspired xanthylium derivatives

Claudia Koch [1,2], Diana-Maria Dreavă [1], Anamaria Todea [1], Francisc Peter [1], Mihai Medeleanu [1], Iulia Păușescu [1*], Corina Samoilă [2,3], Ioan Ovidiu Sîrbu [2,3*]

[1] Politehnica University Timişoara, Faculty of Industrial Chemistry and Environmental Engineering, Carol Telbisz 6, 300001 Timisoara, Romania

[2] Biochemistry Department, University of Medicine and Pharmacy "Victor Babeş", Faculty of Pharmacy, Eftimie Murgu 2,300041 Timisoara, Romania

[3] Center for Complex Networks Science, "Victor Babes" University of Medicine and Pharmacy, Eftimie Murgu 2, 300041 Timisoara, Romania

* Correspondence: iulia.pausescu@upt.ro, ovidiu.sirbu@umft.ro

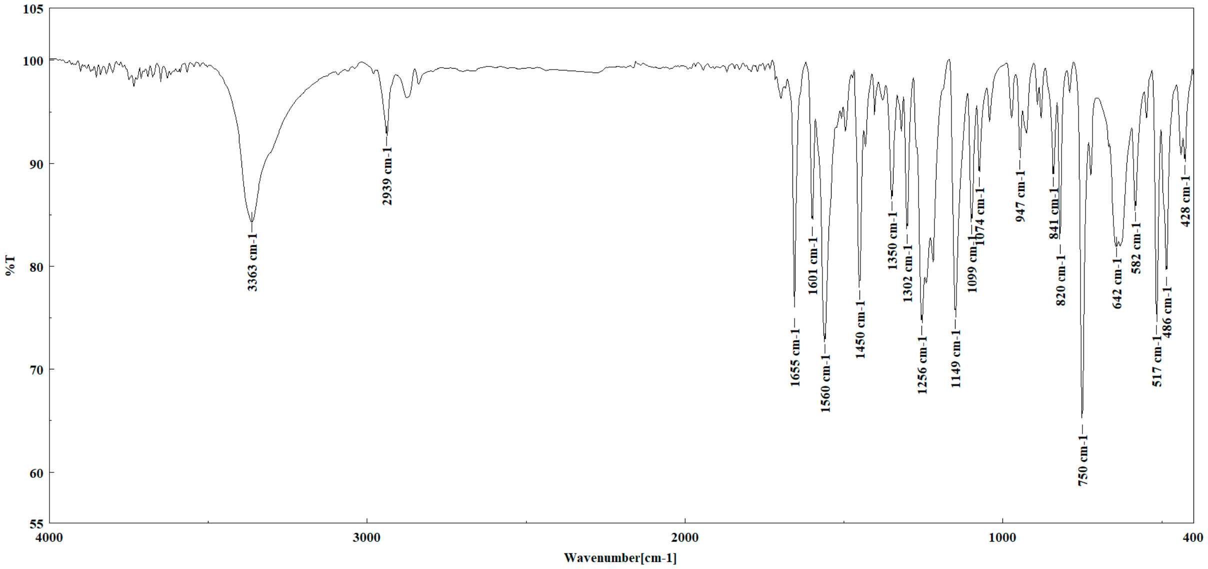

Figure S1. FT-IR spectra of compound **1**

FTIR (ATR) cm^{-1}: 3363, 2939, 1655, 1601, 1560, 1450, 1350, 1302, 1256, 1149, 1099, 1074, 947, 841, 820, 750, 642, 582, 517, 486, 428.

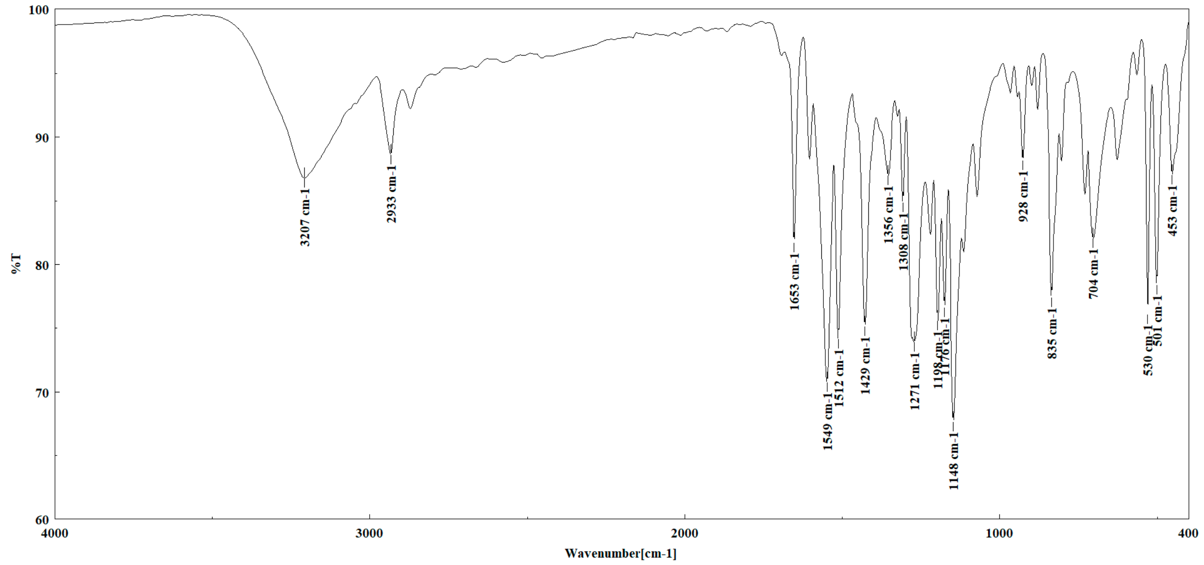

Figure S2. FT-IR spectra of compound **2**

FTIR (ATR) cm^{-1}: 3207, 2933, 1653, 1549, 1429, 1356, 1308, 1271, 1198, 1176, 1148, 928, 835, 704, 530, 501, 453.

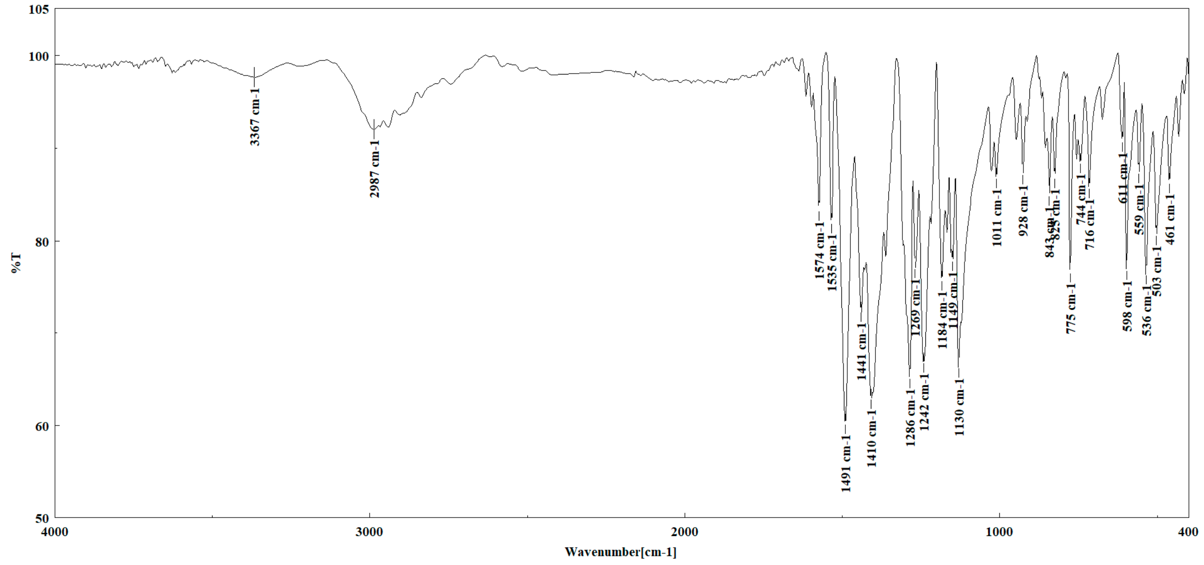

Figure S3. FT-IR spectra of compound **3**

FT-IR (ATR) cm^{-1}: 3367, 2987, 1574, 1535, 1491, 1441, 1410, 1286, 1269, 1242, 1184, 1149, 1130, 1011, 928, 843, 825, 775, 744, 716, 611, 598, 536, 503, 461.

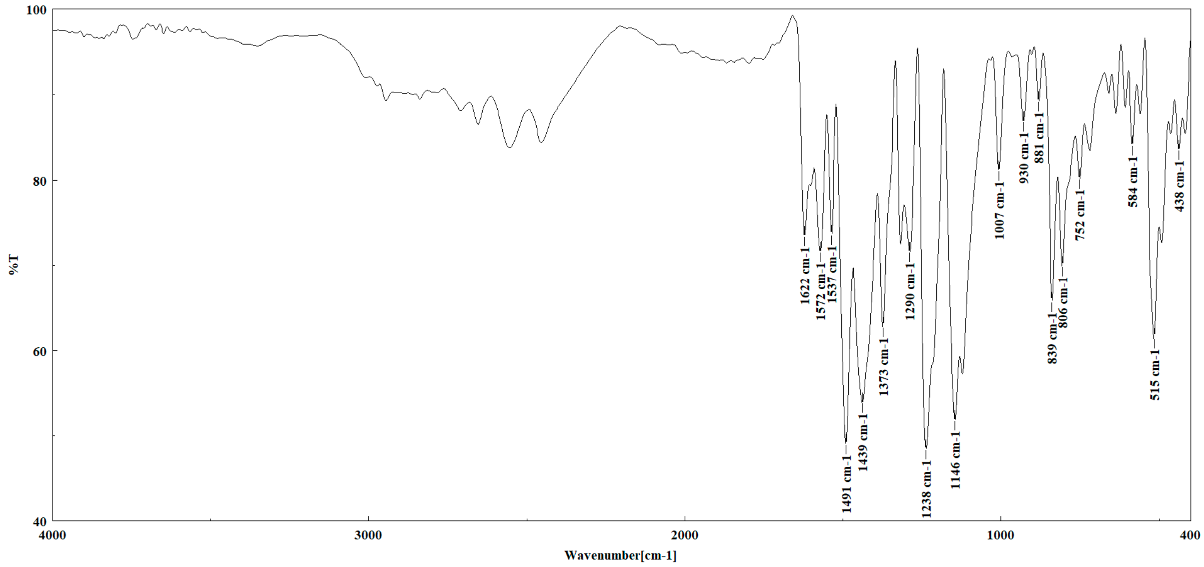

Figure S4. FT-IR spectra of compound **4**

FT-IR (ATR) cm^{-1}: 1622, 1572, 1537, 1491, 1439, 1373, 1290, 1238, 1146, 1007, 930, 839, 806, 752, 584, 515, 438.

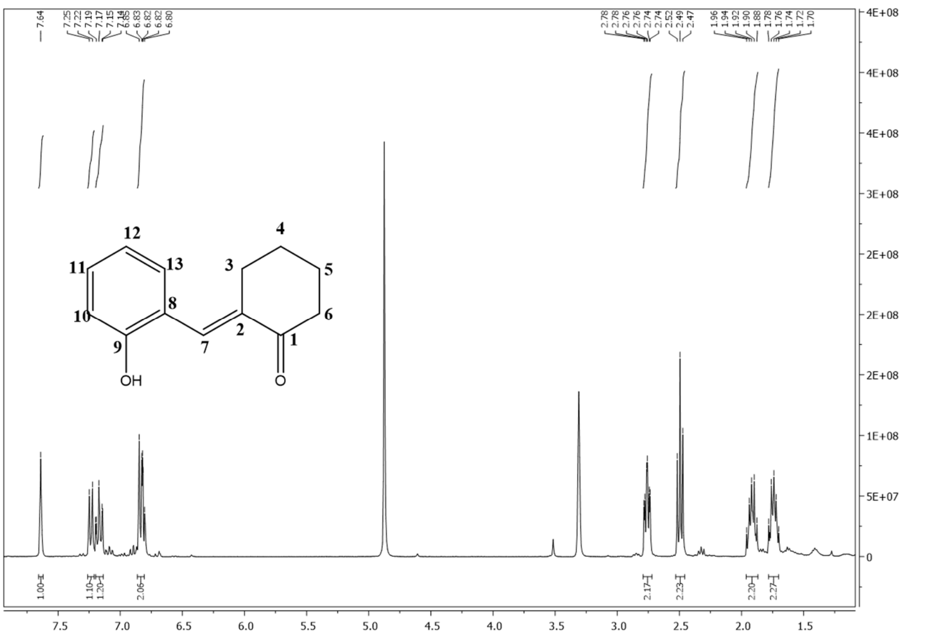

Figure S5. ^{1}**H NMR** spectra of compound **1**

^{1}**H NMR** (300 MHz, MeOD, δ ppm): 7.64 (s, 1H, H7), 7.24 (d, *J* = 7.7 Hz, 1H, H13), 7.16 (dd, *J* = 11.6, 3.9 Hz, 1H, H11), 6.83 (dd, *J* = 5.5, 3.8 Hz, 2H, H10, H12), 2.76 (td, *J* = 6.5, 2.1 Hz, 2H, H6), 2.50 (t, *J* = 6.7 Hz, 2H, H3), 1.92 (dt, *J* = 12.9, 6.5 Hz, 2H, H5), 1.75 (dd, *J* = 12.1, 5.9 Hz, 2H, H4).

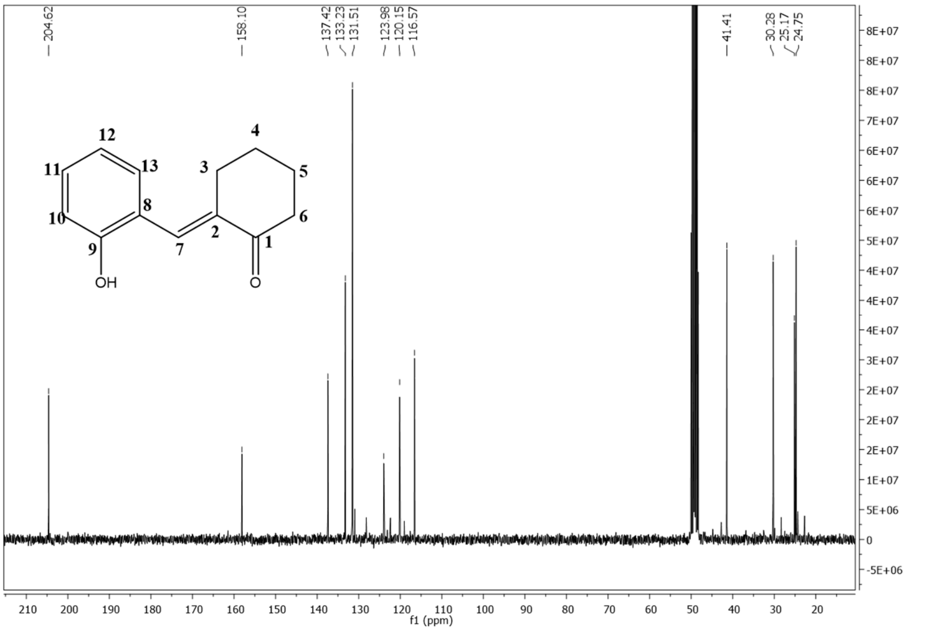

Figure S6. ^{13}**C NMR** spectra of compound **1**

^{13}**C-NMR** (75 MHz, CD_3OD, δ ppm): 204.6 (C1); 158.1 (C9); 137.4 (C2); 133.2 (C7); 131.5 (C11, C13); 123.9 (C8); 120.1 (C12); 116.5 (C10); 41.4 (C6); 30.2 (C3); 25.1 (C4); 24.7 (C5).

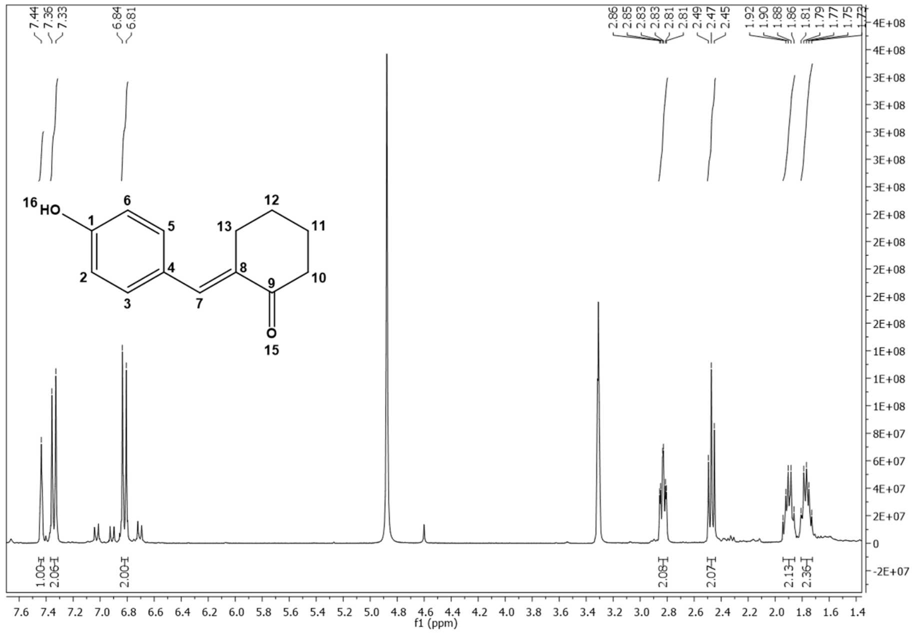

Figure S7. [1]**H NMR** spectra of compound **2**

[1]**H NMR** (300 MHz, MeOD, δ ppm): 7.44 (s, 1H, H7), 7.34 (d, *J* = 8.7 Hz, 2H, H3, H5), 6.82 (d, *J* = 8.7 Hz, 2H, H2, H6), 2.83 (td, *J* = 6.6, 2.1 Hz, 2H, H10), 2.47 (t, *J* = 6.7 Hz, 2H, H13), 1.89 (dd, *J* = 12.2, 6.0 Hz, 2H, H11), 1.76 (dd, *J* = 11.5, 6.2 Hz, 2H, H12).

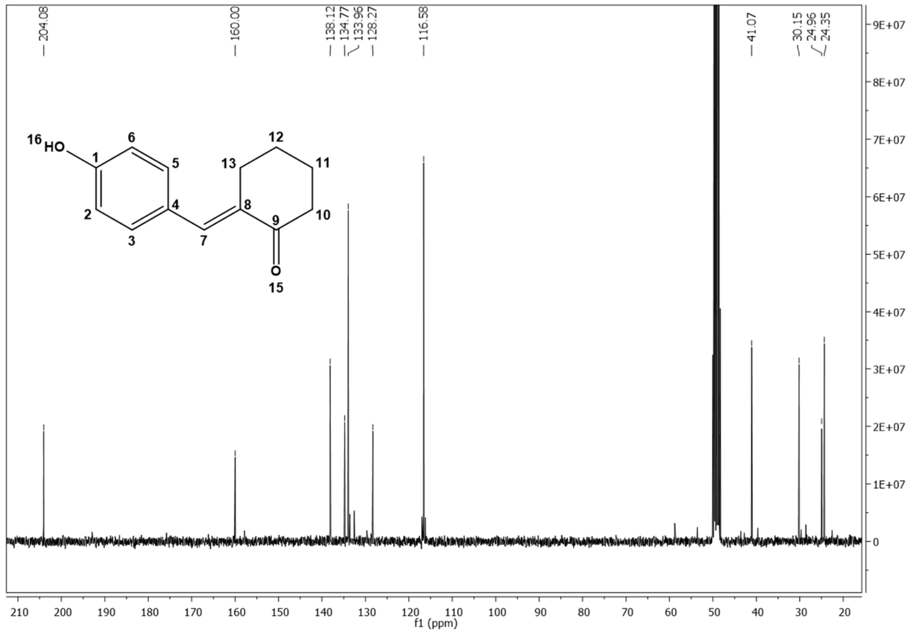

Figure S8. [13]**C NMR** spectra of compound **2**

[13]**C-NMR** (75 MHz, MeOD, δ ppm): 204.0 (C9); 160.0 (C1); 138.1 (C7); 134.7 (C8); 133.9 (C3, C5); 128.2 (C4); 116.5 (C2, C6); 41.0 (C13); 30.1 (C10); 24.9 (C12); 24.3 (C11).

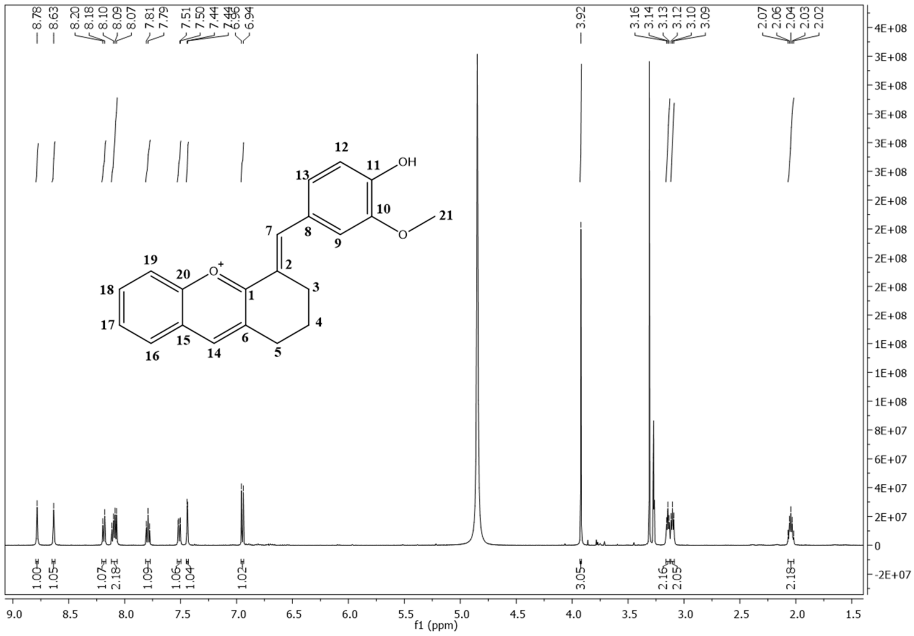

Figure S9. **^{1}H NMR** spectra of compound **3**

^{1}H NMR (500 MHz, MeOD, δ ppm): 8.78 (s, 1H, H14), 8.63 (s, 1H, H7), 8.19 (d, *J* = 8.6 Hz, 1H, H19), 8.09 (dd, *J* = 13.9, 6.9 Hz, 2H, H16, H18), 7.79 (t, *J* = 7.6 Hz, 1H, H17), 7.51 (dd, *J* = 8.4, 1.7 Hz, 1H, H13), 7.44 (d, *J* = 1.7 Hz, 1H, H9), 6.95 (d, *J* = 8.4 Hz, 1H,12), 3.92 (s, 3H, H21), 3.14 (t, *J* = 5.6 Hz, 2H, H3), 3.12 – 3.09 (m, 2H, H5), 2.07 – 2.02 (m, 2H; H4).

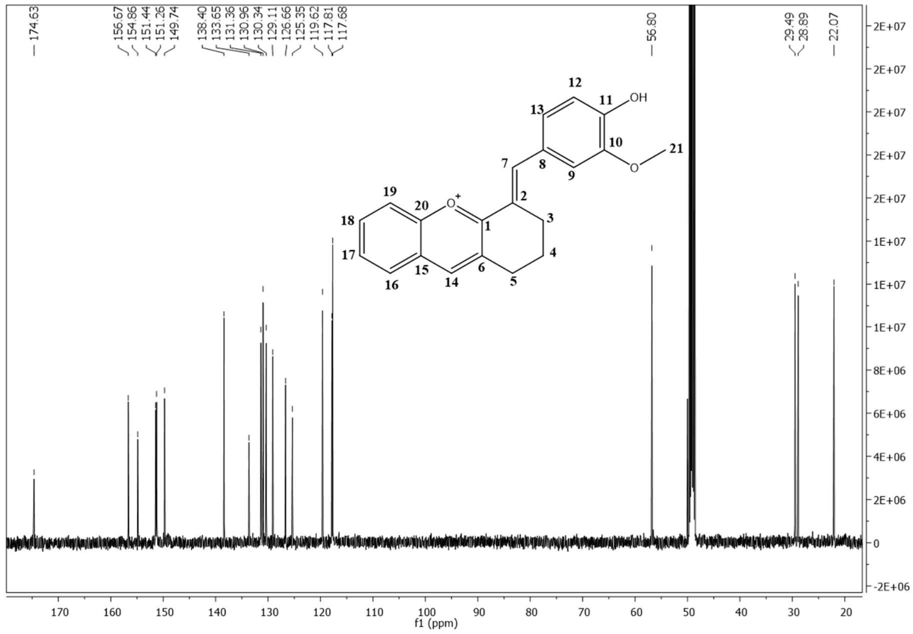

Figure S10. **^{13}C NMR** spectra of compound **3**

^{13}C NMR (125 MHz, MeOD, δ ppm): 174.6 (C1), 156.6 (C20), 154.8 (C11), 151.4 (C14), 151.2 (C7), 149.7 (C10), 138.4 (C18), 133.6 (C6), 131.3 (C13), 130.9 (C16), 130.3 (C17), 129.1 (C8), 126.6 (C2), 125.3 (C15), 119.6 (C19), 117.8 (C9), 117.6 (C12), 56.8 (C21), 29.4 (C3), 28.8 (C5), 22.07 (C4).

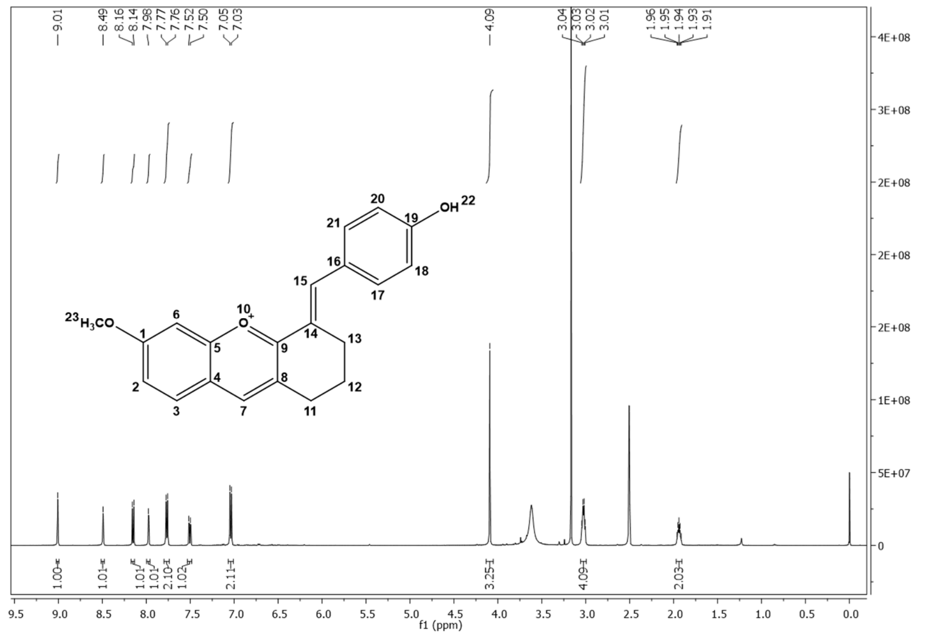

Figure S11. ^{1}**H NMR** spectra of compound **4**

^{1}**H NMR** (500 MHz, DMSO-d_6, δ ppm): 9.01 (s, 1H, H7), 8.49 (s, 1H, H15), 8.15 (d, *J* = 9.0 Hz, 1H, H3), 7.98 (s, 1H, H6), 7.77 (d, *J* = 8.8 Hz, 2H, H17, H21), 7.51 (d, *J* = 9.0 Hz, 1H, H2), 7.04 (d, *J* = 8.7 Hz, 2H, H18, H20), 4.09 (s, 3H, H23), 3.03 (dd, *J* = 12.5, 6.5 Hz, 4H, H11, H13), 1.97 – 1.91 (m, 2H, H12).

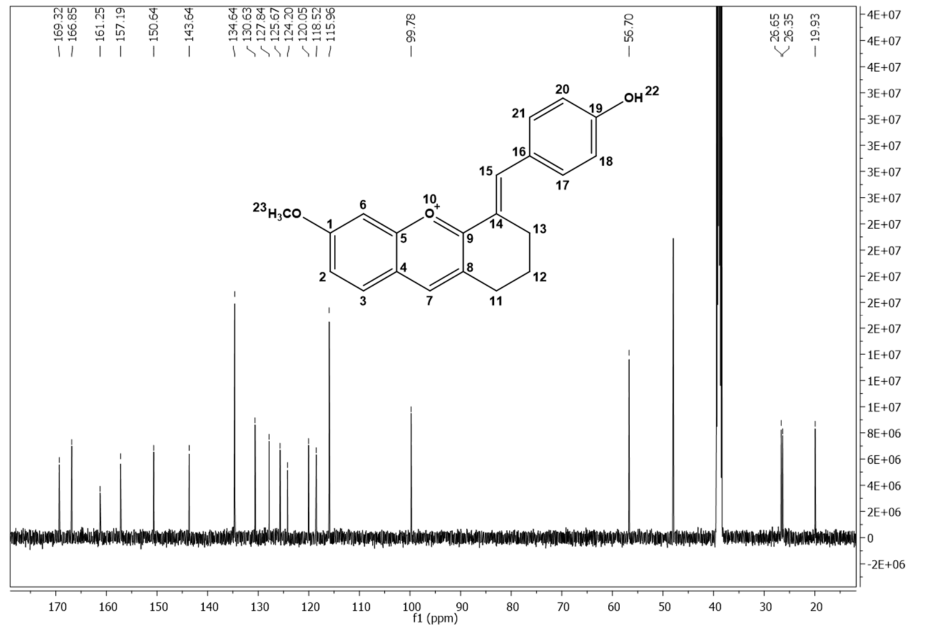

Figure S12. ^{13}**C NMR** spectra of compound **4**

^{13}**C NMR** (125 MHz, DMSO-d_6, δ ppm): 169.3 (C9), 166.8 (C1), 161.2 (C19), 157.1 (C5), 150.6 (C7), 143.6 (C15), 134.6 (C17, C21), 130.6 (C3), 127.8 (C8), 125.6 (C16), 124.2 (C14), 120.0 (C2), 118.5 (C40, 115.9 (C18, C20), 99.7 (C6), 56.7 (C23), 26.6 (C13), 26.3 (C11), 19.9 (C12).

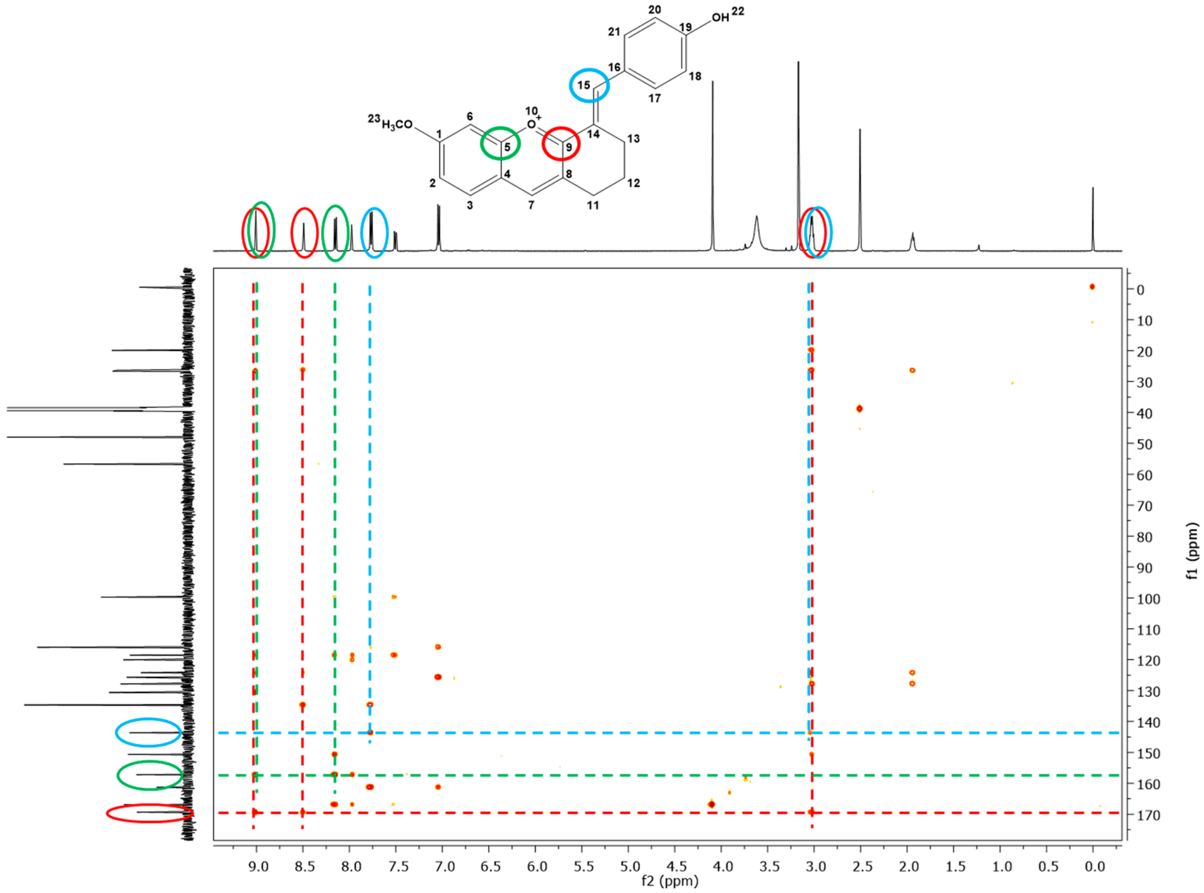

Figure S13. The ^{1}H-^{13}C-HMBC spectrum of compound **4** - the remote couplings between carbon atoms and protons are depicted by different colored circles, green – C5 with H3 and H7, red – C9 with H7, H11, H13 and H15, blue – C15 with H13, H17 and H21.

The formation of compound **4** was further proven by the remote couplings between carbon atoms and protons, shown in the two-dimensional HMBC spectrum. The signal corresponding to C9 carbon atom (169.3 ppm) coupled over two bonds with protons H7 (9.01 ppm), H15 (8.49 ppm), H11 and H13 (3.03 ppm). The remote coupling over two bonds of C5 (157.1 ppm) with protons: H3 (8.15 ppm) and H7 (9.01 ppm) can also be observed. Moreover, the remote coupling of C15 (143.6 ppm) carbon atom with H13 (3.03 ppm), H17 and H21 (7.76 ppm) protons confirmed the formation of compound **4**.
